# Efficacy and safety of perioperative esketamine for postoperative depressive symptoms in breast cancer patients: a meta-analysis

**DOI:** 10.3389/fphar.2026.1806943

**Published:** 2026-07-08

**Authors:** Ruoyi Ji, Xinyuan Shi, Xiaoyang Liu, Hongyi Xiao, Xiaohan Sun, Shiyu Yu, Guimin Dong, Chunhui Zheng, Fanceng Ji

**Affiliations:** 1 School of Psychology and Cognitive Sciences, Peking University, Beijing, China; 2 School of Psychology, Shanghai Jiao Tong University, Shanghai, China; 3 School of Anesthesiology, Shandong Second Medical University, Weifang, China; 4 Medical Imaging College, Shandong Second Medical University, Weifang, China; 5 Department of Anesthesiology, Weifang People’s Hospital, Weifang, China; 6 Breast Disease Center, Weifang People’s Hospital, Weifang, China

**Keywords:** breast cancer, depressive symptoms, esketamine, meta-analysis, perioperative, postoperative pain

## Abstract

**Background:**

Postoperative depressive symptoms may impair recovery and quality of life in patients undergoing breast cancer surgery. Esketamine has shown potential antidepressant and analgesic effects in the perioperative setting. This meta-analysis evaluated the efficacy and safety of perioperative esketamine for postoperative depressive symptoms in patients undergoing breast cancer surgery.

**Methods:**

PubMed, Embase, Cochrane Library, Web of Science, CNKI, and WanFang were systematically searched from database inception to 18 May 2026. Randomized controlled trials comparing perioperative esketamine with control interventions in patients undergoing breast cancer surgery were included. The primary outcome was postoperative depressive symptom score. Secondary outcomes included postoperative pain scores and adverse events. Random-effects models were used as the primary analytical approach. Risk of bias and certainty of evidence were assessed using the RoB 2 tool and the GRADE approach, respectively.

**Results:**

Fifteen randomized controlled trials involving 1,515 patients were included. Perioperative esketamine was associated with significantly lower postoperative depressive symptom scores at postoperative day 1 (SMD = −0.78, 95% CI: −1.08 to −0.48), postoperative day 3 or 48–72 h (SMD = −1.07, 95% CI: −1.63 to −0.51), postoperative day 7 (SMD = −0.79, 95% CI: −1.10 to −0.49), and long-term follow-up (SMD = −1.16, 95% CI: −1.68 to −0.64). For postoperative pain, esketamine was associated with lower postoperative pain scores at postoperative day 1 or 24 h (MD = −0.66, 95% CI: −1.11 to −0.21) and postoperative day 3 or 48–72 h (MD = −0.66, 95% CI: −1.09 to −0.23), but not at postoperative day 7. However, substantial heterogeneity and wide prediction intervals were observed for pain outcomes. The incidences of nausea and vomiting, postoperative delirium, and dizziness did not differ significantly between the esketamine and control groups. The certainty of evidence ranged from low to very low across outcomes.

**Conclusion:**

Perioperative esketamine may reduce postoperative depressive symptom scores and provide short-term analgesic benefits in patients undergoing breast cancer surgery. However, substantial heterogeneity, methodological limitations, and low to very low certainty of evidence warrant cautious interpretation. Further high-quality randomized trials are needed.

**Systematic Review Registration:**

https://www.crd.york.ac.uk/prospero/display_record.php?ID=CRD420251173178, identifier CRD420251173178.

## Introduction

Breast cancer is one of the most common malignant tumors in women worldwide, and its incidence continues to impose a substantial burden on patients and healthcare systems (Wei et al., 2023). Surgery remains a cornerstone of curative treatment for breast cancer. However, breast cancer surgery is not only a physical stressor but also a major psychological challenge. Surgical trauma, perioperative pain, changes in body image, adjuvant treatment, and financial or social burden may all contribute to postoperative mood disturbances (Wang et al., 2020). Among these psychological problems, postoperative depressive symptoms are particularly important because they may impair recovery, reduce quality of life, decrease treatment adherence, prolong hospitalization, and adversely affect long-term prognosis (Kim et al., 2017; Wei et al., 2025). Postoperative pain may also interact with depressive symptoms. Acute postoperative pain, inflammatory activation, and perioperative stress responses may increase vulnerability to depressive symptoms after surgery (Ghoneim and O’Hara, 2016). Conversely, depressive symptoms may intensify pain perception and impair postoperative rehabilitation. This bidirectional relationship suggests that an intervention with both analgesic and antidepressant properties may be particularly relevant in the perioperative management of patients undergoing breast cancer surgery.

Esketamine is the S-enantiomer of racemic ketamine and acts primarily as a noncompetitive N-methyl-D-aspartate receptor antagonist. Compared with racemic ketamine, esketamine has greater affinity for the NMDA receptor and has been used at subanesthetic doses in perioperative anesthesia and analgesia. In addition to its analgesic effects, esketamine has attracted attention because of its rapid antidepressant properties (Zanos and Gould, 2018). Potential mechanisms may involve modulation of glutamatergic neurotransmission, enhancement of brain-derived neurotrophic factor signaling, activation of synaptic plasticity-related pathways such as mTOR, and regulation of stress- and inflammation-related neurobiological processes (Autry et al., 2011; Lou et al., 2024; Xu et al., 2025). However, esketamine may also be associated with adverse effects, including dizziness, nausea and vomiting, psychotomimetic symptoms, hemodynamic changes, and neurocognitive concerns, making safety evaluation essential in the perioperative setting. In recent years, several clinical studies have explored the positive impact of esketamine on the emotional state of patients during the perioperative period of breast cancer, revealing that it can reduce postoperative depressive symptom scores and mitigate opioid-related adverse effects (Guo et al., 2024; Shen et al., 2025). However, current related studies are predominantly single-center, small-sample trials, and their conclusions require further integration and validation. Therefore, this study aims to evaluate the impact of perioperative esketamine on postoperative depressive symptoms in the perioperative period of breast cancer through meta-analysis.

## Materials and methods

This study followed the Preferred Reporting Items for Systematic Reviews and Meta-Analyses (PRISMA) guidelines and was registered in PROSPERO (Registration number: CRD420251173178). The completed PRISMA 2020 checklist is provided as Supplementary Table S1.

### Search strategy and selection criteria

Search terms were utilized. The English search strategy primarily included keywords and their combinations such as: Esketamine, S-ketamine, Breast Cancer, Mastectomy, Depression, depressive symptoms, Postoperative Depression, postoperative depressive symptoms, Perioperative, Randomized controlled trial. Search formulas were constructed for combined retrieval. The following databases were searched: PubMed, Embase, Cochrane Central, Web of Science, CNKI, and WanFang Data. The search period was from database inception to 18 May 2026. The complete search strategies for all databases, including Boolean operators, field tags, search dates, and filters or limits, are provided in Supplementary Table S2. Two researchers screened the titles and abstracts of all initially identified articles. And resolved disagreements through discussion or consultation with a third researcher.

Inclusion Criteria: (1) human randomized controlled trials; (2) patients undergoing breast cancer surgery; (3) perioperative administration of esketamine for treatment or prevention of postoperative depressive symptoms in the experimental group; (4) comparison with normal saline, placebo, or other non-esketamine control interventions; and (5) extractable data for postoperative depressive symptom scores, postoperative pain scores, or adverse events.

Exclusion Criteria: (1) case reports, retrospective studies, review articles, meta-analyses, conference abstracts, dissertations, study protocols, basic research articles, or non-randomized prospective studies; (2) ongoing studies; (3) duplicate publications across databases; (4) studies without a relevant experimental or control group; and (5) studies with incomplete, unavailable, or non-extractable outcome data.

### Data extraction and risk of bias assessment

Data were extracted directly from eligible articles, including first author, publication year, sample size, patient baseline characteristics, surgical type, esketamine administration regimen (dose, timing, method, and duration), control intervention, depressive symptom and pain assessment tools and time points, adverse event incidence, baseline depressive and anxiety symptom scores, history of mood disorders, use of antidepressants, anxiolytics, or other psychotropic medications, and psychiatric disease- or psychotropic medication-related eligibility criteria. Data extraction was performed independently by two researchers, with disagreements resolved through discussion or consultation with a third researcher. Risk of bias was assessed using the revised Cochrane risk-of-bias tool for randomized trials (RoB 2). The following domains were evaluated: bias arising from the randomization process, bias due to deviations from intended interventions, bias due to missing outcome data, bias in measurement of the outcome, and bias in selection of the reported result. Each study was judged as having low risk of bias, some concerns, or high risk of bias. Disagreements were resolved by discussion with a third investigator to reach consensus.

### Statistical analysis

Statistical analyses were performed using R software, version 4.5.3, with the meta package. Continuous outcomes were pooled as standardized mean differences (SMDs) or mean differences (MDs) with 95% confidence intervals (CIs), as appropriate. Because postoperative depressive symptoms were assessed using different psychometric instruments across studies, including the PHQ-9, HAMD-17, MADRS, and SDS, SMDs were calculated for depressive symptom outcomes. Depressive symptom outcomes were analyzed at postoperative day (POD) 1, POD3/48–72 h, POD7, and long-term follow-up. Long-term follow-up was defined as the latest available postoperative depressive symptom assessment at ≥30 days after surgery. In the included studies, this corresponded to POD30, 1 month, POD42, week 12, or 3 months. For postoperative pain outcomes, MDs with 95% CIs were calculated because pain intensity was assessed using comparable 0–10 scales, including the Visual Analogue Scale and Numeric Rating Scale. Pain outcomes were analyzed at POD1 or 24 h, POD3/48–72 h, and POD7. Dichotomous safety outcomes were pooled as risk ratios (RRs) with 95% CIs. Safety outcomes included nausea and vomiting, postoperative delirium, dizziness, and other adverse events when sufficient data were available.

Given the expected clinical and methodological heterogeneity across studies, random-effects models were used as the primary analytical approach. Statistical heterogeneity was assessed using Cochran’s Q test, the I^2^ statistic, and between-study variance (τ^2^). I^2^ values greater than 50% were considered to indicate substantial heterogeneity. Prediction intervals were calculated for random-effects meta-analyses when appropriate to estimate the expected range of treatment effects in future settings.

Exploratory subgroup analyses were conducted to investigate potential sources of heterogeneity. Subgroups were defined according to initial esketamine dose, publication language, and administration regimen. Esketamine dose was categorized as low dose (<0.5 mg/kg) or high dose (≥0.5 mg/kg). Administration regimen was categorized as single or short intraoperative dosing versus continuous, perioperative, or postoperative administration. Continuous, perioperative, or postoperative regimen was defined as esketamine administration involving continuous intraoperative infusion, combined perioperative bolus-plus-infusion protocols, or postoperative continuation through PCIA/analgesic pumps. Single or short intraoperative dose was defined as one-time bolus or short intraoperative infusion without postoperative esketamine continuation. Between-subgroup differences were assessed using tests for subgroup interaction.

Leave-one-out sensitivity analyses were performed by sequentially excluding each individual study to evaluate the robustness of pooled estimates for the main depressive symptom and pain outcomes. Publication bias and small-study effects were assessed using funnel plots and Egger’s regression test when at least 10 studies were available for an outcome. A two-sided P value < 0.05 was considered statistically significant. The certainty of evidence for each outcome was assessed using the GRADE approach and rated as high, moderate, low, or very low. The risk-of-bias domain in the GRADE assessment was informed by the updated RoB 2 judgments. Downgrading was considered for risk of bias, inconsistency, indirectness, imprecision, and publication bias. The full GRADE evidence profile is provided in Supplementary Table S3 (Guyatt et al., 2011).

## Results

### Literature screening process and characteristics of included studies

**FIGURE 1:**
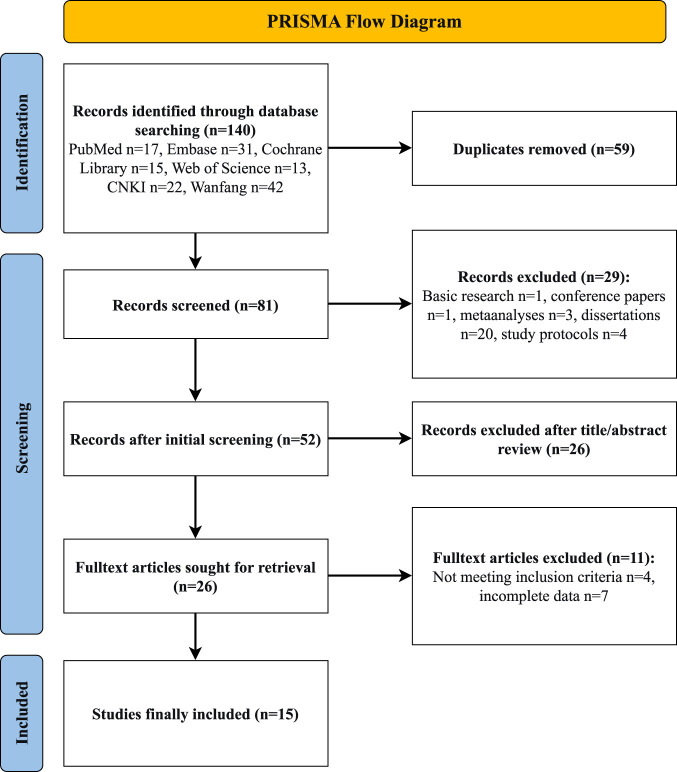
PRISMA flow diagram of study selection. A total of 140 records were identified through database searching. After removal of 59 duplicates, 81 records were screened. Following title, abstract, and full-text assessment, 15 studies were ultimately included in the meta-analysis.

A total of 140 records were identified through database searching, including PubMed (n = 17), Embase (n = 31), Cochrane Library (n = 15), Web of Science (n = 13), CNKI (n = 22), and WanFang (n = 42). After removal of 59 duplicate records, 81 records remained. Of these, 29 records were excluded because they were basic research articles (n = 1), conference papers (n = 1), meta-analyses (n = 3), dissertations (n = 20), or study protocols (n = 4). The remaining 52 records underwent title and abstract screening, after which 26 records were excluded. Twenty-six full-text articles were sought for retrieval and assessed for eligibility. Of these, 11 articles were excluded because they did not meet the inclusion criteria (n = 4) or had incomplete data (n = 7). Ultimately, 15 studies involving 1,515 patients were included in this meta-analysis. The flowchart is shown in Figure 1, and the characteristics of the included studies are summarized in Table 1.

**TABLE 1:** Characteristics of the included studies.

First author/Year	Blinding	Sample size (experimental/Control)	Patient age (years)	Surgical type	Esketamine administration method	Eske-tamine dose	Control group treatment	Depression Rating scale	Pain Rating scale	Preop depression score	Antidep. Use	Postoperative depression follow-up time	Postoperative pain follow-up time	Postoperative adverse event type	Postop analgesic use
Huanwei Wang 2024	Double blind	32/32	42.3 ± 5.8/41.8 ± 5.8	Unilateral modified radical mastectomy	Single dose	0.2 mg/kg	0.2 mL/kg normal saline	PHQ-9	VAS	3.28 ± 3.15/3.22 ± 3.44	No	Preoperative, POD 1, 3, 7, 30	POD 1, day 3, day 7	Nausea, vomiting, delirium	Yes; flurbiprofen, tramadol PRN
Peirong Liu 2021	Double blind	101/100 (S-ketamine/control; three-arm trial)	48.0 ± 10.2/46.6 ± 8.2	Modified radical mastectomy for breast cancer	Single dose	0.125 mg/kg	Same volume saline	HAMD-17	VAS	16.8 ± 2.3/17.0 ± 2.2/17.0 ± 2.2	No	Baseline, POD 3, 1 week, 1 month, 3 months	POD 1, day 3	Nausea, vomiting, dizziness	Yes; PCIA ropivacaine + fentanyl
Qingfeng Wei 2025	Double blind	52/52 (mITT)	51.0 (46.0–55.0)/51.0 (46.75–58.0)	Elective breast cancer resection	Infusion over 40 min	0.25 mg/kg	0.9% saline	MADRS	NRS	20.0(17.0–23.25)/21.0(17.0–24.0)	No	POD 1–5, week 4, week 12	48 h postoperative	Nausea, vomiting, dizziness, delirium, diplopia	Yes; NSAIDs + fentanyl
Mengting Shen 2025	Double blind	40/40	53.8 ± 9.4/54.8 ± 8.4	Unilateral modified radical mastectomy	Bolus + continuous infusion	0.5 mg/kg + 0.25 mg/kg/h	Normal saline bolus + infusion	HAMD-17, SDS	VAS	HAMD: 5.0(4.0–7.8)/4.5(3.0–7.0) SDS: 39.5(32.9–43.1)/35.5(31.1–44.1)	No	POD 1, POD 3, POD 30	30 min after extubation, POD 1, POD 2	Psychotomimetic symptoms, dizziness, agitation, PONV	Yes; sufentanil + flurbiprofen PRN
Song-Yuan Liu 2026	Double blind	60/60	54 (45–65)/51 (45–56)	Breast-conserving surgery; modified radical mastectomy; simple mastectomy with SLNB	Bolus + continuous infusion	0.2 mg/kg + 0.1 mg/kg/h	0.9% sodium chloride	PHQ-9	NRS	5.3 ± 3.70/5.2 ± 2.44	NR	Baseline, POD 7, POD 30	POD 1	Nausea, vomiting, drowsiness, psychiatric symptoms	NR; pain assessed
Mingxia Li 2023	Unclear	30/30	54 ± 9/54 ± 8	Breast cancer surgery	PCIA	50 mg in PCIA	PCIA with sufentanil alone	SDS	VAS	51 ± 5/50 ± 5	NR	Preoperative day 1, POD 3	POD 1, POD 2	Dizziness, nausea/vomiting, hypotension	Yes; parecoxib + PCIA
Qin Pan 2024	Unclear	48/47	51.60 ± 6.60/51.68 ± 6.61	Radical mastectomy for breast cancer	Single dose + thoracic nerve block	0.5 mg/kg	Same volume saline + nerve block	SDS	VAS	69.34 ± 4.27/67.33 ± 4.34	NR	Preoperative, 24 h postoperative	1, 6, 12, 48 h	Nausea, vomiting, hypotension, arrhythmia	Yes; PCA sufentanil
Jiapeng Wang 2024	Unclear	33/33	57.03 ± 4.06/54.36 ± 8.43	Radical mastectomy for breast cancer	Bolus + infusion + PCIA	0.1 mg/kg, 0.1 mg/kg/h, 1 mg/kg	Nerve block without esketamine	HAMD-17	VAS	14.06 ± 1.54/15.00 ± 1.58	No	Preoperative, POD 3, 7, 1 month	POD 1, day 3, day 7	Nausea, vomiting	Yes; PCIA sufentanil + flurbiprofen
Yuanzheng Xu 2023	Single blind	43/43	48.89 ± 5.57/48.16 ± 5.53	Radical or modified radical mastectomy for breast cancer	Single dose	0.2 mg/kg	Routine anesthesia/saline	SDS	Not reported	46.66 ± 7.26/46.25 ± 7.20	NR	Preoperative, POD 1, POD 3	Not reported	Cognitive dysfunction, delirium, nausea/vomiting	Yes; IV analgesia pump
Tao Ji 2024	Single blind	24/24/24/24 (four groups)	50.3 ± 6.8/49.6 ± 5.4/49.0 ± 8.7/51.2 ± 8.4	Modified radical mastectomy for breast cancer	Single dose	0.15/0.30/0.50 mg/kg	Same volume saline	MADRS	Not reported	15.3 ± 5.4/16.7 ± 7.0/17.0 ± 6.0/15.6 ± 5.5	No	Preoperative, POD 1, 3, 5, 7	Not reported	Delirium, PONV, dizziness/drowsiness, fever	Yes; rescue analgesia recorded
Junyong Zhu 2022	Double blind	40/40	46.7 ± 8.7/47.6 ± 8.6	Radical mastectomy for breast cancer	Single dose + postoperative analgesia pump	0.125 mg/kg + 0.125 mg/kg within 48 h	Same volume saline	HAMD-17	VAS	4.38 ± 2.03/4.23 ± 2.11	NR	Preoperative, POD 2, POD 7	1 h after extubation, 24 h	Postoperative adverse reactions	Yes; PCIA sufentanil ± S-ket.
Shuangshuang He 2025	Unclear	40/40	50.67 ± 6.83/50.07 ± 6.21	Modified radical mastectomy for breast cancer	Continuous infusion + PCIA	0.25–0.5 mg/kg; 15 μg/kg/h	Sufentanil-based regimen	HAMD-17	VAS	14.78 ± 3.21/14.38 ± 3.08	NR	POD 1, POD 3, POD 7	POD 1, POD 3, POD 7	Nausea, vomiting, dizziness, pruritus	Yes; PCIA (esket. Or sufentanil)
Tingyun Wang 2026	Double blind	58/58/58 (low/high/control)	50.6 ± 9.2/52.1 ± 8.5/51.3 ± 8.7	Breast cancer resection (total or breast-conserving)	Continuous infusion	0.2 or 0.5 mg/kg	Normal saline placebo	HAMD-17, PHQ-9	VAS	15.1 ± 3.0/14.9 ± 3.3/14.8 ± 3.2	No	POD 1, POD 7, POD 42	POD 1, POD 3, POD 7	Respiratory depression, nausea, vomiting	Yes; flurbiprofen PRN
Zhiru Zhao 2025	Unclear	57/57	51 ± 4/50 ± 5	Radical mastectomy for breast cancer	Bolus + continuous infusion	0.5 mg/kg + 0.25 mg/(kg·h)	Same volume saline	HAMD	NRS	7.8 ± 1.2/7.8 ± 1.5	NR	Preoperative, POD 1, POD 3, POD 7	6, 12, 24, 48 h	Blurred vision, dizziness, nausea/vomiting, hallucination	Yes; PCIA sufentanil + flurbiprofen
Hui Ding 2024	Double blind	48/47 (analyzed)	67.6 ± 7.8/67.1 ± 7.2	Modified radical mastectomy for breast cancer (elderly)	Bolus + continuous infusion	0.25 mg/kg + 0.125 mg/(kg·h)	Equal-volume normal saline	HAMD-17	Not reported	14(8)/15(9)	NR	POD 3, POD 7	Not reported	Hypotension, tachycardia, delayed neurocognitive recovery	Yes; PCIA sufentanil 48 h

Values are presented as mean ± SD, median (interquartile range), number/number, or as reported in the original studies. Antidep. use indicates preoperative or perioperative antidepressant or psychotropic medication use, as reported in each study. HAMD-17, 17-item Hamilton Depression Rating Scale; MADRS, Montgomery–Åsberg Depression Rating Scale; mITT, modified intention-to-treat; NR, not reported; NRS, Numeric Rating Scale; NSAIDs, nonsteroidal anti-inflammatory drugs; PCA, patient-controlled analgesia; PCIA, patient-controlled intravenous analgesia; PHQ-9, Patient Health Questionnaire-9; POD, postoperative day; PONV, postoperative nausea and vomiting; PRN, pro re nata/as needed; SDS, Self-Rating Depression Scale; SLNB, sentinel lymph node biopsy; VAS, Visual Analogue Scale.

All included studies were randomized controlled trials, with total study sample sizes ranging from 60 to 201 participants. Eight studies used a double-blind design, two studies used a single-blind design, and five studies did not clearly report the blinding method. The included patients underwent breast cancer–related surgical procedures, mainly radical mastectomy, modified radical mastectomy, breast-conserving surgery, or breast cancer resection.

For postoperative depressive symptom assessment, eight studies (Shen et al., 2025; Liu et al., 2021; Wang JP. et al., 2024; Zhu et al., 2022; He et al., 2025; Wang et al., 2026; Zhao et al., 2025; Ding et al., 2024) used the Hamilton Depression Rating Scale, including HAMD or HAMD-17, four studies (Shen et al., 2025; Li et al., 2023; Pan et al., 2024; Xu et al., 2023) used the Self-Rating Depression Scale, three studies (Wang et al., 2026; Wang H. et al., 2024; Liu et al., 2026) used the Patient Health Questionnaire-9, and two studies (Wei et al., 2025; Ji et al., 2024) used the Montgomery-Åsberg Depression Rating Scale. Some studies reported more than one depressive-symptom scale. In addition, several studies reported anxiety-related scales, including the Self-Rating Anxiety Scale, State Anxiety Inventory, and Beck Anxiety Inventory. For postoperative pain assessment, nine studies used the Visual Analogue Scale, three studies used the Numeric Rating Scale, and three studies did not report a pain rating scale. The postoperative follow-up periods varied across studies, ranging from immediate or early postoperative assessments, such as 1 h after extubation and 6–48 h after surgery, to later follow-up points including postoperative day 30, week 12, and 3 months. Regarding preoperative antidepressant use, seven studies explicitly excluded or did not use related antidepressant/psychotropic drugs, while eight studies did not clearly report this information. In terms of postoperative analgesia, except for one study that did not clearly specify the postoperative analgesic regimen, all other studies employed postoperative analgesia measures, mainly including patient-controlled intravenous analgesia (PCIA), sufentanil, fentanyl, flurbiprofen axetil, parecoxib, tramadol, or nonsteroidal anti-inflammatory drugs (NSAIDs). The adverse events reported in the included studies mainly included nausea and vomiting, dizziness, delirium, hallucinations, psychiatric symptoms, agitation, hypotension, tachycardia, respiratory depression, and cognitive dysfunction.

### Risk of bias assessment

**FIGURE 2:**
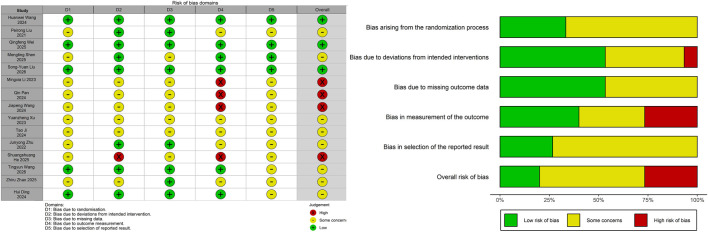
Risk-of-bias assessment of the included randomized trials using RoB 2. Risk of bias was assessed using the revised Cochrane risk-of-bias tool for randomized trials (RoB 2). The five domains were: D1, bias arising from the randomization process; D2, bias due to deviations from intended interventions; D3, bias due to missing outcome data; D4, bias in measurement of the outcome; and D5, bias in selection of the reported result. Each domain and the overall judgment were classified as low risk of bias, some concerns, or high risk of bias. The left panel shows domain-level and overall judgments for each study, and the right panel summarizes the distribution of judgments across domains.

The results of the risk-of-bias assessment using RoB 2 are shown in Figure 2. All 15 included randomized trials were assessed across the five RoB 2 domains, and each domain-level judgment and overall judgment was classified as low risk of bias, some concerns, or high risk of bias. Overall, three studies were judged as having low risk of bias, eight studies as having some concerns, and four studies as having high risk of bias.

Across individual RoB 2 domains, the lowest risk was observed for bias due to missing outcome data, for which eight studies were judged as low risk and seven studies as having some concerns, with no study judged as high risk. Bias due to deviations from intended interventions was judged as low risk in eight studies, as some concerns in six studies, and as high risk in one study. Bias arising from the randomization process was judged as low risk in five studies and as some concerns in ten studies. Bias in selection of the reported result was judged as low risk in four studies and as some concerns in eleven studies. Bias in measurement of the outcome was the domain with the greatest methodological concern, with six studies judged as low risk, five as having some concerns, and four as having high risk of bias. The high-risk judgments were mainly driven by concerns in outcome measurement and, in one study, deviations from intended interventions. These findings indicate that although several trials had acceptable methodological quality, the overall body of evidence had important risk-of-bias concerns.

### Depressive symptom outcomes

A total of four meta-analyses were conducted to evaluate the effect of esketamine on postoperative depressive symptoms at different follow-up time points.

**FIGURE 3:**
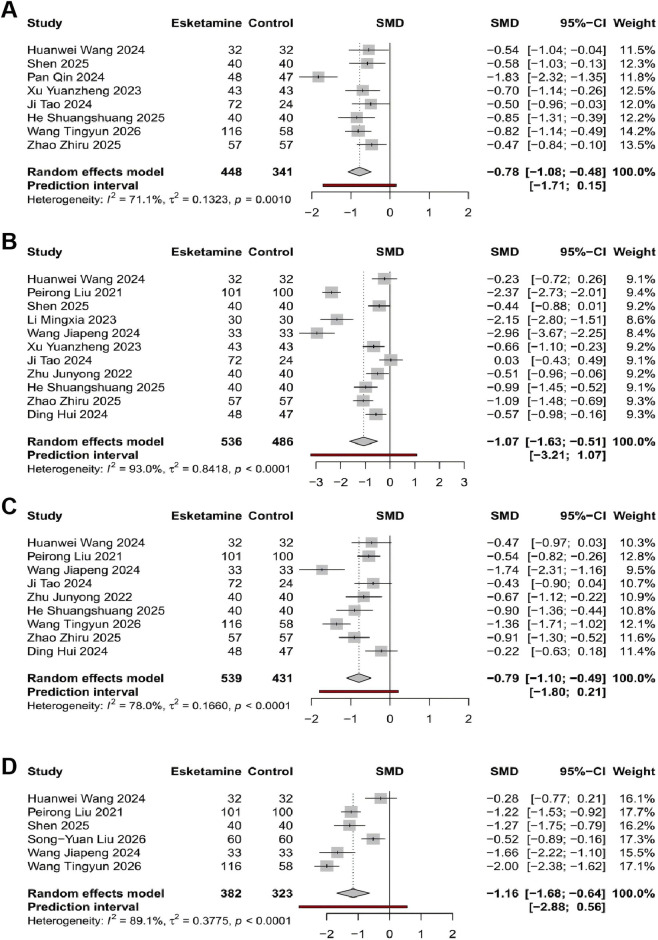
Forest plots of the effect of perioperative esketamine on postoperative depressive symptom scores. Panels show pooled standardized mean differences for depressive symptom scores at POD1 **(A)**, POD3/48–72 h **(B)**, POD7 **(C)**, and long-term follow-up **(D)**. Random-effects models were used. Negative SMD values favor esketamine over control. CI, confidence interval; POD, postoperative day; SMD, standardized mean difference.

At POD1, eight studies involving 789 patients were included. The pooled analysis showed that esketamine significantly reduced depressive symptom scores compared with the control group (SMD = −0.78, 95% CI: −1.08 to −0.48), with moderate to substantial heterogeneity (I^2^ = 71.1%, p = 0.0010) (Figure 3A).

At POD3/48–72 h, 11 studies involving 1,022 patients were included. Esketamine was associated with significantly lower depressive symptom scores than the control intervention (SMD = −1.07, 95% CI: −1.63 to −0.51). Considerable heterogeneity was observed (I^2^ = 93.0%, p < 0.0001) (Figure 3B).

At POD7, nine studies involving 970 patients were included. The pooled result indicated that esketamine significantly reduced depressive symptom scores (SMD = −0.79, 95% CI: −1.10 to −0.49), with substantial heterogeneity (I^2^ = 78.0%, p < 0.0001) (Figure 3C).

For long-term follow-up, six studies involving 705 patients were analyzed. The pooled analysis demonstrated that esketamine significantly reduced depressive symptom scores compared with the control group (SMD = −1.16, 95% CI: −1.68 to −0.64), although marked heterogeneity remained (I^2^ = 89.1%, p < 0.0001) (Figure 3D).

Overall, these findings suggest that perioperative esketamine may improve postoperative depressive symptoms across multiple postoperative time points.

### Postoperative pain scores

**FIGURE 4:**
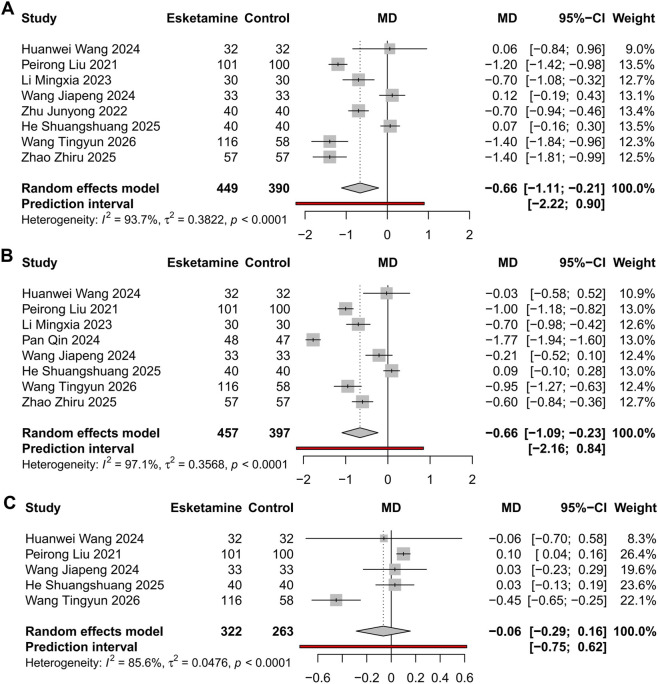
Forest plots of the effect of perioperative esketamine on postoperative pain scores. Panels show pooled mean differences in postoperative pain scores at POD1 or 24 h **(A)**, POD3/48–72 h **(B)**, and POD7 **(C)**. Random-effects models were used. Negative MD values indicate lower pain scores in the esketamine group. CI, confidence interval; MD, mean difference; POD, postoperative day; VAS, Visual Analogue Scale.

Eight studies involving 839 patients reported pain scores at POD1 or 24 h after surgery. The pooled analysis showed that esketamine was associated with significantly lower postoperative pain scores compared with the control group (MD = −0.66, 95% CI: −1.11 to −0.21, P = 0.0041). However, substantial heterogeneity was observed among studies (I^2^ = 93.7%, τ^2^ = 0.3822, P < 0.0001), and the prediction interval crossed the null effect (−2.22 to 0.90) (Figure 4A).

Eight studies involving 854 patients reported pain scores at POD3/48–72 h after surgery. The pooled result also favored esketamine, showing significantly lower pain scores than the control intervention (MD = −0.66, 95% CI: −1.09 to −0.23, P = 0.0024). Considerable heterogeneity was detected (I^2^ = 97.1%, τ^2^ = 0.3568, P < 0.0001), and the prediction interval was wide and crossed the null effect (−2.16 to 0.84) (Figure 4B).

Five studies involving 585 patients reported pain scores at POD7. No significant difference was observed between the esketamine and control groups (MD = −0.06, 95% CI: −0.29 to 0.16). Heterogeneity remained substantial (I^2^ = 85.6%, τ^2^ = 0.0476, P < 0.0001) (Figure 4C).

Overall, perioperative esketamine was associated with lower pain scores at POD1/24 h and POD3/48–72 h, but not at POD7. Given the substantial heterogeneity and wide prediction intervals, these analgesic findings should be interpreted cautiously.

### Adverse events

**FIGURE 5:**
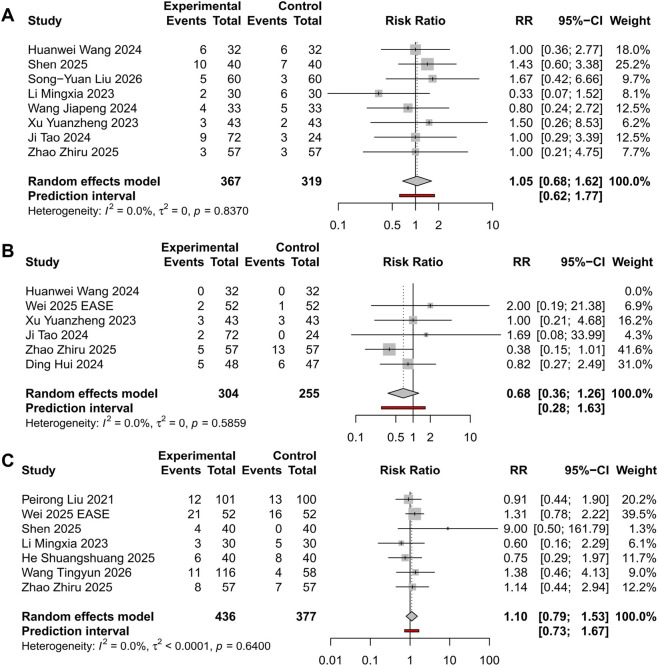
Forest plots of adverse events associated with perioperative esketamine. Panels show pooled risk ratios for nausea and vomiting **(A)**, postoperative delirium **(B)**, and dizziness **(C)**. Random-effects models were used. RR values below one favor esketamine, whereas values above one favor control. CI, confidence interval; RR, risk ratio.

Eight studies involving 686 patients reported nausea and vomiting. The pooled analysis showed no significant difference between the esketamine and control groups in the incidence of nausea and vomiting (RR = 1.05, 95% CI: 0.68 to 1.62, P = 0.828). No heterogeneity was detected among studies (I^2^ = 0.0%, τ^2^ = 0, P = 0.8370) (Figure 5A).

Six studies involving 559 patients reported postoperative delirium. The pooled result showed that esketamine was not associated with a significant difference in the incidence of postoperative delirium compared with the control group (RR = 0.68, 95% CI: 0.36 to 1.26, P = 0.218). No heterogeneity was observed (I^2^ = 0.0%, τ^2^ = 0, P = 0.5859) (Figure 5B).

Seven studies involving 813 patients reported dizziness. The incidence of dizziness did not differ significantly between the esketamine and control groups (RR = 1.10, 95% CI: 0.79 to 1.53, P = 0.560), with no evidence of heterogeneity (I^2^ = 0.0%, τ^2^ < 0.0001, P = 0.6400) (Figure 5C).

Overall, perioperative esketamine was not associated with a statistically significant increase in the risk of nausea and vomiting, postoperative delirium, or dizziness. However, the safety findings should be interpreted cautiously because adverse events were not consistently reported across all included studies and the number of events was limited for some outcomes.

### Subgroup analyses results

#### Depressive symptoms

Exploratory subgroup analyses were performed to investigate potential sources of heterogeneity.

**FIGURE 6:**
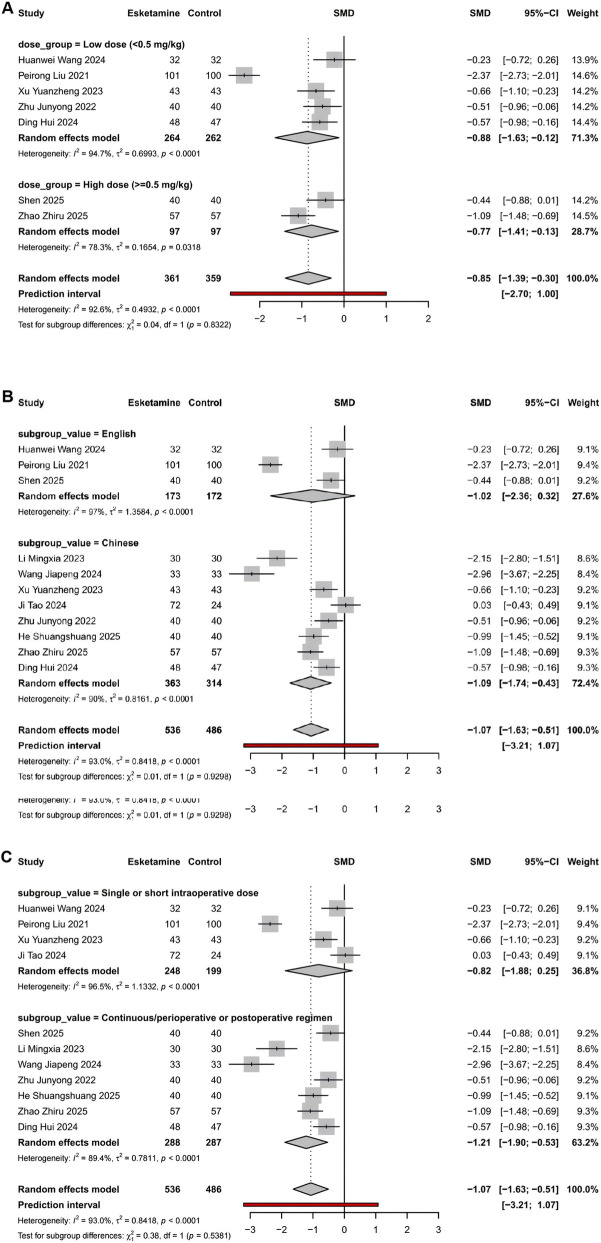
Subgroup analyses of depressive symptom scores at POD3/48–72 h. Forest plots show subgroup analyses stratified by esketamine dose category **(A)**, publication language **(B)**, and administration regimen **(C)**. Random-effects models were used. Negative SMD values favor esketamine. CI, confidence interval; POD, postoperative day; SMD, standardized mean difference.

For depressive symptom scores at POD3/48–72 h, subgroup analysis by initial esketamine dose showed that both low-dose esketamine and high-dose esketamine were associated with lower depressive symptom scores. In the low-dose subgroup, the pooled effect was SMD = −0.88, 95% CI: −1.63 to −0.12, with substantial heterogeneity (I^2^ = 94.7%). In the high-dose subgroup, the pooled effect was SMD = −0.77, 95% CI: −1.41 to −0.13, with substantial heterogeneity (I^2^ = 78.3%). The test for subgroup differences was not statistically significant (P = 0.8322), indicating no evidence of a dose-dependent subgroup effect (Figure 6A).

When stratified by publication language, the pooled effect was SMD = −1.02, 95% CI: −2.36 to 0.32 in English-language studies and SMD = −1.09, 95% CI: −1.74 to −0.43 in Chinese-language studies. The between-subgroup difference was not significant (P = 0.9298), suggesting that publication language did not explain the observed heterogeneity (Figure 6B).

Subgroup analysis according to administration regimen showed that the pooled effect was SMD = −0.82, 95% CI: −1.88 to 0.25 in studies using a single or short intraoperative dose and SMD = −1.21, 95% CI: −1.90 to −0.53 in studies using continuous, perioperative, or postoperative regimens. The test for subgroup differences was not statistically significant (P = 0.5381), indicating no clear evidence that administration regimen modified the antidepressant effect at POD3/48–72 h (Figure 6C).

#### Pain

**FIGURE 7:**
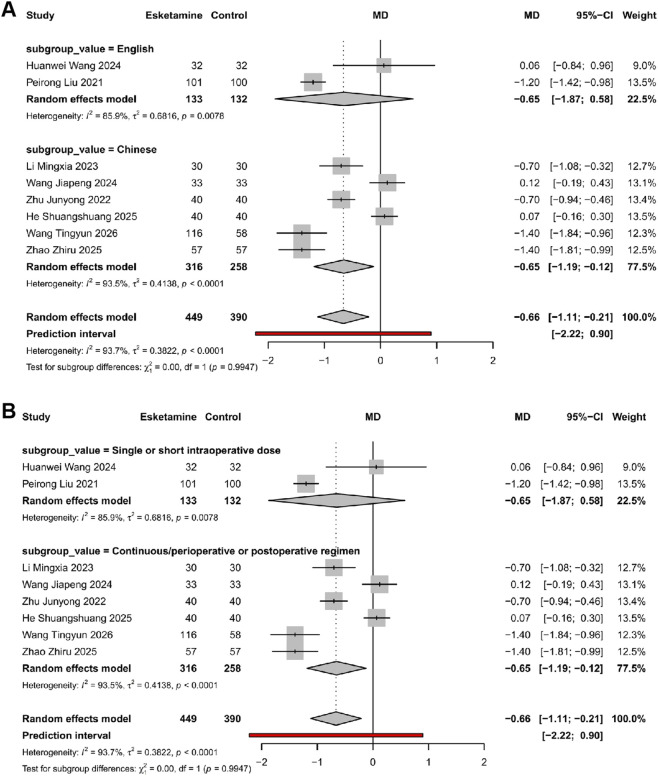
Subgroup analyses of postoperative pain scores at POD1 or 24 h. Forest plots show subgroup analyses stratified by publication language **(A)** and administration regimen **(B)**. Random-effects models were used. Negative MD values indicate lower pain scores in the esketamine group. CI, confidence interval; MD, mean difference; POD, postoperative day.

For pain scores at POD1/24 h, subgroup analysis by publication language showed similar pooled effects in English-language studies (MD = −0.65, 95% CI: −1.87 to 0.58) and Chinese-language studies (MD = −0.65, 95% CI: −1.19 to −0.12). The test for subgroup differences was not significant (P = 0.9947) (Figure 7A).

Similarly, subgroup analysis by administration regimen for POD1/24 h pain scores showed no significant subgroup difference (P = 0.9947). The pooled effect was MD = −0.65, 95% CI: −1.87 to 0.58 in the single or short intraoperative dose subgroup and MD = −0.65, 95% CI: −1.19 to −0.12 in the continuous, perioperative, or postoperative regimen subgroup (Figure 7B).

Overall, none of the subgroup analyses demonstrated statistically significant between-subgroup differences. Therefore, dose, publication language, and administration regimen did not clearly explain the substantial heterogeneity observed in the main analyses.

#### Publication bias

**FIGURE 8:**
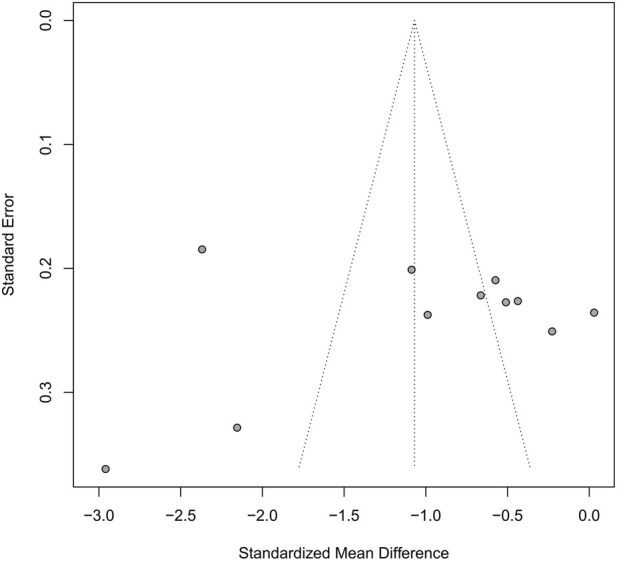
Funnel plot for depressive symptom scores at POD3/48–72 h. The funnel plot was used to assess potential publication bias or small-study effects for depressive symptom scores at POD3/48–72 h. Egger’s regression test did not indicate significant funnel plot asymmetry. SMD, standardized mean difference.

A funnel plot and Egger’s regression test were used to assess potential publication bias or small-study effects for depressive symptom scores at POD3/48–72 h, which was the only outcome with at least 10 included studies. No clear evidence of marked asymmetry was observed, and Egger’s regression test did not indicate significant small-study effects (P = 0.6325) (Figure 8).

#### Sensitivity analysis

Leave-one-out sensitivity analyses were performed by sequentially omitting each individual study to evaluate the robustness of the pooled results.

**FIGURE 9:**
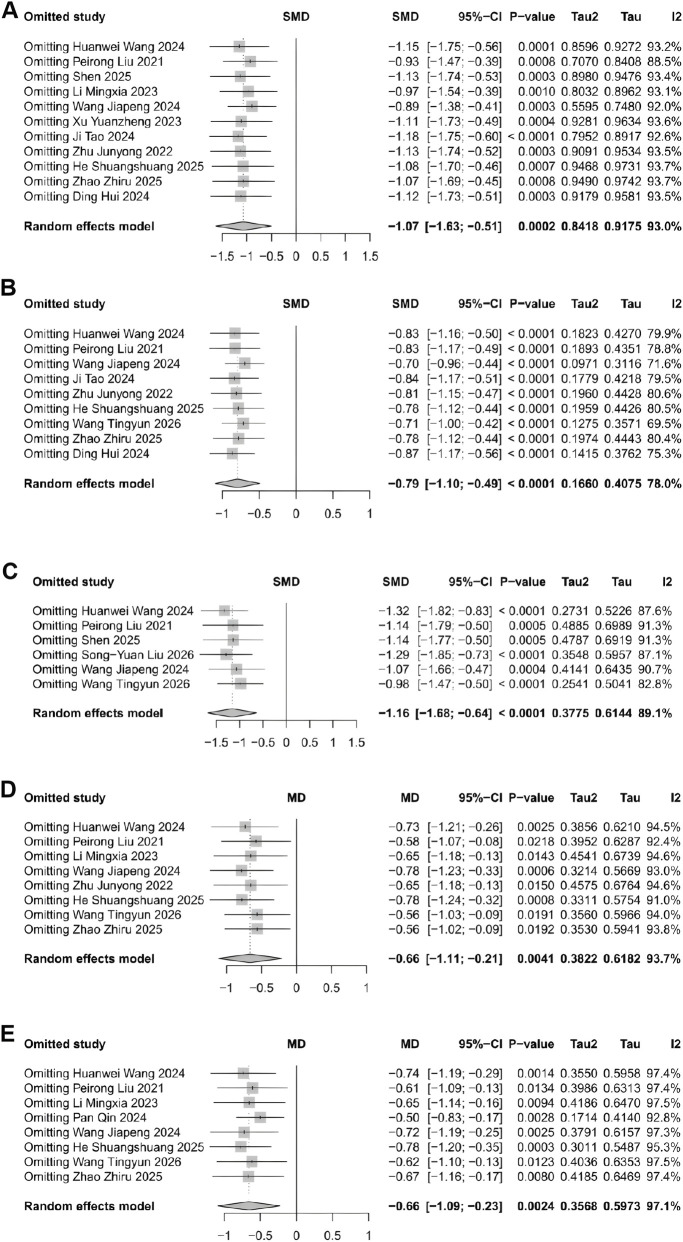
Leave-one-out sensitivity analyses of depressive symptom and pain outcomes. Sensitivity analyses were performed by sequentially omitting each individual study. Panels show results for depressive symptom scores at POD3/48–72 h **(A)**, POD7 **(B)**, and long-term follow-up **(C)**, as well as postoperative pain scores at POD1 or 24 h **(D)** and POD3/48–72 h **(E)**. CI, confidence interval; MD, mean difference; SMD, standardized mean difference; VAS, Visual Analogue Scale.

For depressive symptom scores at POD3/48–72 h, the pooled effect remained statistically significant after exclusion of each individual study, with recalculated SMDs ranging from −1.18 to −0.89. The direction of effect consistently favored esketamine, suggesting that the overall result was not driven by any single study (Figure 9A).

For depressive symptom scores at POD7, the pooled estimates also remained stable after omitting each study, with SMDs ranging from −0.87 to −0.70. All recalculated estimates remained statistically significant and favored esketamine (Figure 9B).

For long-term depressive symptom scores, exclusion of individual studies did not materially alter the pooled effect. The recalculated SMDs ranged from −1.32 to −0.98, and all estimates remained statistically significant (Figure 9C).

Sensitivity analyses for pain outcomes showed similar robustness. For POD1/24 h pain scores, the pooled MDs after omitting individual studies ranged from −0.78 to −0.56 (Figure 9D). For POD3/48–72 h pain scores, the pooled MDs ranged from −0.78 to −0.50, with all estimates continuing to favor esketamine (Figure 9E). And all recalculated estimates remained statistically significant.

Overall, these sensitivity analyses indicated that the main findings for depressive symptom scores and early postoperative pain scores were not substantially influenced by any single study.

#### Certainty of evidence

The certainty of evidence was assessed using the GRADE approach and is summarized in Supplementary Table S3. The risk-of-bias domain in the GRADE assessment was informed by the updated RoB 2 judgments. Overall, the certainty of evidence ranged from low to very low across outcomes.

For Depressive symptom outcomes, the certainty of evidence was rated as low for depressive symptom scores at POD1, POD3/48–72 h, and POD7, and very low for long-term depressive symptom scores. The main reasons for downgrading were RoB 2-derived serious risk of bias and substantial or marked inconsistency across studies.

For pain outcomes, the certainty of evidence was rated as very low for both POD1/24 h and POD3/48–72 h pain scores. These outcomes were downgraded because of RoB 2-derived serious risk of bias and very serious inconsistency. The prediction intervals for both pain outcomes crossed the null effect, indicating substantial uncertainty regarding the expected effect in future similar studies.

For adverse events, the certainty of evidence was rated as low for nausea and vomiting and dizziness, and very low for postoperative delirium. These outcomes were downgraded mainly because of RoB 2-derived serious risk of bias and imprecision. No serious indirectness was identified for the included outcomes. Publication bias was not detected for depressive symptom scores at POD3/48–72 h based on Egger’s test, whereas publication bias could not be reliably assessed for several other outcomes because fewer than 10 studies were included.

## Discussion

This meta-analysis of 15 randomized controlled trials involving 1,515 patients evaluated the efficacy and safety of perioperative esketamine for postoperative depressive symptoms in patients undergoing breast cancer surgery. The main findings suggest that perioperative esketamine may reduce postoperative depressive symptom scores from POD1 to long-term follow-up. Esketamine was also associated with lower postoperative pain scores at POD1/24 h and POD3/48–72 h, but not at POD7. However, substantial heterogeneity and wide prediction intervals were observed for pain outcomes, indicating that the analgesic findings should be interpreted cautiously. For safety outcomes, perioperative esketamine was not associated with a statistically significant increase in nausea and vomiting, postoperative delirium, or dizziness.

The results of this meta-analysis suggest that perioperative esketamine may reduce postoperative depressive symptom scores in patients undergoing breast cancer surgery. This effect may be partly explained by its pharmacological action as an NMDA receptor antagonist and by downstream neuroplastic mechanisms. Previous studies have suggested that ketamine and esketamine may promote synaptic plasticity through pathways involving brain-derived neurotrophic factor (BDNF), mTOR signaling, and synaptogenesis, which may contribute to rapid antidepressant effects (Autry et al., 2011; Li et al., 2010; Yang et al., 2015). In addition, emerging evidence indicates that the antidepressant effects of ketamine may involve mechanisms beyond NMDA receptor blockade. For example, adenosine signaling has recently been proposed as a potential pathway through which ketamine may produce antidepressant effects (Yue et al., 2026). Breast cancer surgery is both a physical and psychological stressor, and perioperative stress may activate inflammatory responses and the hypothalamic-pituitary-adrenal axis, thereby increasing susceptibility to depressive symptoms (Schumacher, 2024). Early perioperative administration of esketamine may modulate these stress-related neurobiological pathways after surgery (Salahudeen et al., 2020; Dai et al., 2025). However, whether the observed reductions in postoperative depressive symptom scores translate into prevention of persistent depressive disorders remains uncertain.

Postoperative pain is also clinically relevant when interpreting the potential effect of perioperative esketamine on postoperative depressive symptoms. Acute and chronic postoperative pain may contribute to the development or worsening of depressive symptoms, and the relationship between pain and depressive symptoms is likely bidirectional (Yu et al., 2022). Esketamine may provide analgesic effects through NMDA receptor antagonism and inhibition of central sensitization, which provides a plausible biological rationale for its use in the perioperative setting (Chen et al., 2023; Bahji et al., 2021). In the present meta-analysis, perioperative esketamine was associated with lower pain scores at POD1/24 h and POD3/48–72 h, whereas no significant difference was observed at POD7. Nevertheless, these findings should be interpreted cautiously because heterogeneity was substantial and the prediction intervals crossed the null effect. Therefore, although esketamine may provide short-term reductions in postoperative pain scores, the current evidence is insufficient to confirm a stable or clinically generalizable analgesic effect in this population. Further trials are needed to determine whether perioperative esketamine can meaningfully reduce postoperative pain intensity and whether such analgesic effects contribute to longer-term psychological recovery (Miziara et al., 2016; Popova et al., 2019).

Exploratory subgroup analyses were performed to investigate potential sources of heterogeneity. For depressive symptom scores at POD3/48–72 h, both low-dose esketamine (<0.5 mg/kg) and high-dose esketamine (≥0.5 mg/kg) showed effect estimates favoring esketamine, but the test for subgroup differences was not statistically significant. This finding does not support a clear dose-dependent subgroup effect. Subgroup analysis by publication language also showed no significant between-subgroup difference, suggesting that publication language did not explain the observed heterogeneity. Similarly, administration regimen did not significantly modify the antidepressant effect, although the pooled estimate appeared numerically greater in studies using continuous, perioperative, or postoperative regimens than in those using a single or short intraoperative dose. For POD1/24 h pain scores, subgroup analyses by publication language and administration regimen also did not show significant between-subgroup differences. These analyses suggest that initial dose, publication language, and administration regimen did not clearly account for the substantial heterogeneity observed in the main analyses. The optimal dose, timing, and duration of perioperative esketamine administration therefore remain uncertain. Future dose-finding trials are needed to clarify whether specific administration strategies provide more consistent antidepressant or analgesic benefits.

The adverse event analysis showed no statistically significant differences between the esketamine and control groups in nausea and vomiting, postoperative delirium, or dizziness. These findings suggest that perioperative esketamine was not associated with an increased risk of the commonly pooled adverse events in the included trials. However, the safety findings should be interpreted cautiously because adverse events were inconsistently reported across studies, and the number of events was limited for several outcomes. Some previous perioperative studies have suggested that esketamine may reduce opioid requirements, which could theoretically influence opioid-related adverse effects such as postoperative nausea and vomiting (Su et al., 2022; Hou et al., 2025). However, opioid consumption and analgesic rescue data were not consistently available in the included trials. Therefore, the relationship between esketamine, opioid-sparing effects, and postoperative adverse events could not be directly examined in this meta-analysis.

This meta-analysis has several limitations. First, substantial heterogeneity was observed across most depressive symptom and pain outcomes. This heterogeneity may have arisen from differences in patient baseline characteristics, preoperative psychological status, depressive symptom rating scales, surgical procedures, anesthesia protocols, esketamine dose, timing and duration of administration, postoperative analgesic regimens, and follow-up time points. Although subgroup analyses were performed, the limited number of studies in each subgroup prevented a comprehensive explanation of heterogeneity. Second, depressive symptoms were measured using different psychometric instruments, including HAMD or HAMD-17, SDS, PHQ-9, and MADRS. Although standardized mean differences were used to account for different scales, this approach limits direct clinical interpretability. Third, most included studies had relatively small sample sizes, and several studies had methodological limitations and were judged as having some concerns or high risk of bias according to RoB 2. Fourth, the available evidence was largely derived from Chinese populations or Chinese-language publications, which may limit the generalizability of the findings to other healthcare systems and ethnic populations. Fifth, adverse events were not uniformly defined or consistently reported across trials, and some safety outcomes had limited event counts. Finally, baseline psychological status was not consistently assessed or reported across the included trials. Although several studies measured preoperative or baseline depressive or anxiety symptoms, the presence of clinically diagnosed mood disorders, previous psychiatric history, and baseline use of antidepressants, anxiolytics, or other psychotropic medications were not uniformly reported. Therefore, it remains unclear whether perioperative esketamine is more effective in patients with pre-existing depressive symptoms, diagnosed mood disorders, or no baseline mood disturbance. The potential influence of concomitant psychotropic medication use on postoperative depressive symptom outcomes also could not be evaluated.

## Conclusion

Perioperative esketamine may reduce postoperative depressive symptom scores in patients undergoing breast cancer surgery and may provide short-term postoperative analgesic benefits. Current evidence does not suggest a significant increase in nausea and vomiting, postoperative delirium, or dizziness. However, because of substantial heterogeneity, methodological limitations, and low to very low certainty of evidence, these findings should be interpreted cautiously. Further high-quality randomized controlled trials are required to confirm the efficacy and safety of perioperative esketamine in this population.

## Data Availability

The original contributions presented in the study are included in the article/Supplementary Material, further inquiries can be directed to the corresponding author.
