# Supplementary material for: Efficacy and safety of perioperative esketamine for postoperative depressive symptoms in breast cancer patients: a meta-analysis
Source: Front Pharmacol. 2026 Jul 8;17:1806943. doi: 10.3389/fphar.2026.1806943 (PMC13389019; doi:10.3389/fphar.2026.1806943)
Supplement: Supplementary file 2 [file Table2.docx]

Supplementary Material: Search Strategies and Records Retrieved

Date of search: May 18, 2026

| Database | Date Searched | Records Retrieved | Full Search String |
| --- | --- | --- | --- |
| **PubMed** | May 18, 2026 | 17 | ("Esketamine"[Mesh] OR "Ketamine"[Mesh] OR esketamine[tiab] OR "S-ketamine"[tiab] OR "S ketamine"[tiab] OR "S(+)-ketamine"[tiab]) AND ("Breast Neoplasms"[Mesh] OR breast cancer[tiab] OR breast carcinoma[tiab] OR breast tumor[tiab] OR mastectomy[tiab] OR "Mastectomy"[Mesh]) AND ("Depression"[Mesh] OR depression[tiab] OR depressive[tiab] OR postoperative depression[tiab] OR post-operative depression[tiab] OR "Postoperative Period"[Mesh]) AND ("Perioperative Period"[Mesh] OR perioperative[tiab] OR peri-operative[tiab] OR intraoperative[tiab] OR intra-operative[tiab] OR postoperative[tiab] OR post-operative[tiab]) AND ("Randomized Controlled Trial"[Publication Type] OR randomized controlled trial[tiab] OR randomised controlled trial[tiab] OR RCT[tiab] OR controlled clinical trial[tiab] OR randomly[tiab] OR placebo[tiab]) |
| **Embase** | May 18, 2026 | 31 | ('esketamine'/exp OR 'ketamine'/exp OR esketamine:ab,ti OR 's-ketamine':ab,ti OR 's ketamine':ab,ti OR 's(+)-ketamine':ab,ti) AND ('breast cancer'/exp OR 'breast cancer':ab,ti OR 'breast carcinoma':ab,ti OR 'breast tumor':ab,ti OR mastectomy:ab,ti OR 'mastectomy'/exp) AND ('depression'/exp OR depression:ab,ti OR depressive:ab,ti OR 'postoperative depression':ab,ti OR 'post-operative depression':ab,ti) AND ('perioperative period'/exp OR perioperative:ab,ti OR 'peri-operative':ab,ti OR intraoperative:ab,ti OR 'intra-operative':ab,ti OR postoperative:ab,ti OR 'post-operative':ab,ti) AND ('randomized controlled trial'/exp OR 'randomized controlled trial':ab,ti OR 'randomised controlled trial':ab,ti OR rct:ab,ti OR 'controlled clinical trial':ab,ti OR randomly:ab,ti OR placebo:ab,ti) |
| **Cochrane Central** | May 18, 2026 | 15 | (esketamine OR "S-ketamine" OR "S ketamine" OR "S(+)-ketamine"):ti,ab,kw AND ("breast cancer" OR "breast carcinoma" OR "breast tumor" OR mastectomy):ti,ab,kw AND (depression OR depressive OR "postoperative depression" OR "post-operative depression"):ti,ab,kw AND (perioperative OR "peri-operative" OR intraoperative OR "intra-operative" OR postoperative OR "post-operative"):ti,ab,kw AND ("randomized controlled trial" OR "controlled clinical trial" OR RCT OR randomly OR placebo):ti,ab,kw |
| **Web of Science** | May 18, 2026 | 13 | TS=((esketamine OR "S-ketamine" OR "S ketamine" OR "S(+)-ketamine")) AND TS=(("breast cancer" OR "breast carcinoma" OR "breast tumor" OR mastectomy)) AND TS=((depression OR depressive OR "postoperative depression" OR "post-operative depression")) AND TS=((perioperative OR "peri-operative" OR intraoperative OR "intra-operative" OR postoperative OR "post-operative")) AND TS=(("randomized controlled trial" OR "randomised controlled trial" OR RCT OR "controlled clinical trial" OR randomly OR placebo)) |
| **CNKI (中国知网)** | May 18, 2026 | 22 | (SU='艾司氯胺酮' OR SU='氯胺酮' OR AB='艾司氯胺酮' OR AB='S-氯胺酮' OR AB='右旋氯胺酮') AND (SU='乳腺癌' OR SU='乳腺肿瘤' OR SU='乳房切除术' OR AB='乳腺癌' OR AB='乳腺肿瘤' OR AB='乳房切除术') AND (SU='抑郁' OR SU='术后抑郁' OR AB='抑郁' OR AB='术后抑郁') AND (SU='围术期' OR SU='围手术期' OR SU='术中' OR SU='术后' OR AB='围术期' OR AB='围手术期') AND (SU='随机对照试验' OR SU='随机' OR AB='随机' OR AB='RCT') |
| **WanFang Data (万方)** | May 18, 2026 | 42 | 主题:(艾司氯胺酮 OR 氯胺酮 OR S-氯胺酮) AND 主题:(乳腺癌 OR 乳腺肿瘤 OR 乳房切除术) AND 主题:(抑郁 OR 术后抑郁) AND 主题:(围术期 OR 围手术期 OR 术中 OR 术后) AND 主题:(随机对照试验 OR 随机 OR RCT) |
| **Total before deduplication** | – | 140 | – |
