# Supplementary material for: Efficacy and safety of perioperative esketamine for postoperative depressive symptoms in breast cancer patients: a meta-analysis
Source: Front Pharmacol. 2026 Jul 8;17:1806943. doi: 10.3389/fphar.2026.1806943 (PMC13389019; doi:10.3389/fphar.2026.1806943)
Supplement: Supplementary file 3 [file Table3.docx]

Supplementary Table S3. GRADE evidence profile based on the updated RoB 2 assessment.

| **GRADE evidence profile based on RoB 2 assessment** | | | | | | | | | | | | |
| --- | --- | --- | --- | --- | --- | --- | --- | --- | --- | --- | --- | --- |
| Esketamine for postoperative depression, pain, and adverse events after breast cancer surgery | | | | | | | | | | | | |
| **Outcome** | **No. studies** | **Participants** | **Study design** | **Risk of bias** | **Inconsistency** | **Indirectness** | **Imprecision** | **Publication bias / other considerations** | **Effect estimate (95% CI)** | **Certainty** | **GRADE symbols** | **Main rationale** |
| Depressive symptom score at POD1 (lower is better) | 8 | 789 | Randomized trials | Serious (-1) | Serious (-1) | Not serious | Not serious | Undetected; fewer than 10 studies | SMD -0.78 (-1.08 to -0.48) | **LOW** | ⊕⊕◯◯ | Downgraded for RoB2-derived serious risk of bias and substantial heterogeneity (I² = 71.1%). |
| Depressive symptom score at POD3 or 48-72 h (lower is better) | 11 | 1022 | Randomized trials | Serious (-1) | Serious (-1) | Not serious | Not serious | Not serious; Egger test P = 0.6325 | SMD -1.07 (-1.63 to -0.51) | **LOW** | ⊕⊕◯◯ | Downgraded for RoB2-derived serious risk of bias and substantial heterogeneity (I² = 93.0%). |
| Depressive symptom score at POD7 (lower is better) | 9 | 970 | Randomized trials | Serious (-1) | Serious (-1) | Not serious | Not serious | Undetected; fewer than 10 studies | SMD -0.79 (-1.10 to -0.49) | **LOW** | ⊕⊕◯◯ | Downgraded for RoB2-derived serious risk of bias and substantial heterogeneity (I² = 78.0%). |
| Depressive symptom score at long-term follow-up (lower is better) | 6 | 705 | Randomized trials | Serious (-1) | Very serious (-2) | Not serious | Not serious | Undetected; fewer than 10 studies | SMD -1.16 (-1.68 to -0.64) | **VERY LOW** | ⊕◯◯◯ | Downgraded for RoB2-derived serious risk of bias and marked heterogeneity (I² = 89.1%). |
| Pain score at POD1 or 24 h (lower is better) | 8 | 839 | Randomized trials | Serious (-1) | Very serious (-2) | Not serious | Not serious | Undetected; fewer than 10 studies | MD -0.66 (-1.11 to -0.21) | **VERY LOW** | ⊕◯◯◯ | Downgraded for RoB2-derived serious risk of bias and marked heterogeneity (I² = 93.7%); prediction interval crossed the null effect. |
| Pain score at POD3 or 48-72 h (lower is better) | 8 | 854 | Randomized trials | Serious (-1) | Very serious (-2) | Not serious | Not serious | Undetected; fewer than 10 studies | MD -0.66 (-1.09 to -0.23) | **VERY LOW** | ⊕◯◯◯ | Downgraded for RoB2-derived serious risk of bias and very serious heterogeneity (I² = 97.1%); prediction interval crossed the null effect. |
| Nausea and vomiting | 8 | 686 | Randomized trials | Serious (-1) | Not serious | Not serious | Serious (-1) | Undetected; fewer than 10 studies | RR 1.05 (0.68 to 1.62) | **LOW** | ⊕⊕◯◯ | Downgraded for RoB2-derived serious risk of bias and imprecision; confidence interval includes potential benefit and harm. |
| Dizziness | 7 | 813 | Randomized trials | Serious (-1) | Not serious | Not serious | Serious (-1) | Undetected; fewer than 10 studies | RR 1.10 (0.79 to 1.53) | **LOW** | ⊕⊕◯◯ | Downgraded for RoB2-derived serious risk of bias and imprecision; confidence interval crosses the null effect. |
| Postoperative delirium | 6 | 559 | Randomized trials | Serious (-1) | Not serious | Not serious | Very serious (-2) | Undetected; fewer than 10 studies | RR 0.68 (0.36 to 1.26) | **LOW** | ⊕⊕◯◯ | Downgraded for RoB2-derived serious risk of bias and imprecision; confidence interval includes potential benefit and no effect. |

The certainty of evidence was assessed using the GRADE approach. The risk-of-bias domain was informed by the updated RoB 2 judgments. CI, confidence interval; GRADE, Grading of Recommendations Assessment, Development and Evaluation; MD, mean difference; POD, postoperative day; RR, risk ratio; SMD, standardized mean difference.
